# Protein kinase C in porcine retinal arteries and neuroretina following retinal ischemia-reperfusion

**Published:** 2009-04-13

**Authors:** Bodil Gesslein, Lotta Gustafsson, Angelica Wackenfors, Fredrik Ghosh, Malin Malmsjö

**Affiliations:** 1 Department of Ophthalmology, Lund University, Sweden; 2 Department of Medicine, Lund University, Sweden

## Abstract

**Purpose:**

Identification of the intracellular signal-transduction pathways activated in retinal ischemia may be important in revealing novel pharmacological targets. To date, most studies have focused on identifying neuroprotective agents. The retinal blood vessels are key organs in circulatory failure, and this study was therefore designed to examine the retinal vasculature separately from the neuroretina.

**Methods:**

Retinal ischemia was induced by elevating the intraocular pressure in porcine eyes, followed by 5, 12, or 20 h of reperfusion. Protein kinase C (PKC)α, PKCβ1, and PKCβ2 mRNA levels, and protein expression were determined using real-time PCR, western blot, and immunofluorescence staining techniques.

**Results:**

The retinal arteries could easily be dissected free and studied separately from the neuroretina in this porcine model. The *PKCα*, *PKCβ1*, and *PKCβ2* mRNA levels tended to be lower in ischemia-reperfused than in sham-operated eyes in both the retinal arteries and the neuroretina. This was most prominent after 5 h, and less pronounced after 12 h and 20 h of reperfusion. Likewise, the protein levels of PKCα, PKCβ1, and PKCβ2 were slightly lower following ischemia-reperfusion when compared to sham-operated eyes. PKCα, PKCβ1, and PKCβ2 immunostaining were observed in bipolar cells of the neuroretina and in endothelial cells, and to a low extent in the smooth muscle layer, of the retinal arteries.

**Conclusions:**

Retinal ischemia followed by reperfusion results in lower levels of PKC in both the neuroretina and retinal arteries. New targets for pharmacological treatment may be found by studying the retinal vasculature so as to identify the intracellular signal-transduction pathways involved in the development of injury following retinal circulatory failure.

## Introduction

Retinal ischemia due to local circulatory failure in diabetes, vein thrombosis, and arterial occlusion is a major cause of sight-threatening complications and blindness [1]. In retinal ischemia, new blood vessels are formed to meet the metabolic demands of the ischemic tissue. The newly formed blood vessels malfunction and are unable to replace the flow of necessary nutrients. They leak and bleed and are thus no longer part of the blood–brain barrier. This causes sight-threatening complications such as tractional retinal detachment, vitreous hemorrhage, neovascular glaucoma, and macular edema [1–3]. Retinal ischemia is treated with laser photocoagulation, which is effective in saving vision, but at the expense of large portions of the retina and its photoreceptors. Even though numerous studies, aimed at limiting the extent of retinal injury after ischemia, have been performed, there is still no effective pharmacological treatment for this condition [2,4].

Most studies have focused on identifying neuroprotective agents for the treatment of retinal ischemia-reperfusion injury [1]. The blood vessels of the retina are key organs in local circulation failure, and it may therefore be important not only to examine the neuroretina but also the retinal vasculature. For this purpose we set up and evaluated a porcine model of pressure-induced retinal ischemia in which the retinal arteries could be studied separately from the neuroretina. The porcine eye has previously been proven to be suitable for experimental analysis of the retinal arteries [5–7].

In the field of cerebral and cardiac ischemia, protein kinase C (PKC) has been shown to play a central role [8–11]. Pathological changes in the vasculature during stroke and ischemic heart disease can be reduced by treatment with PKC inhibitors [12–14]. In the eye, PKC levels are altered in several ischemic conditions, including diabetic retinopathy and central vein occlusion [15,16]. However, studies on PKC and retinal ischemia have thus far mainly involved small animals and rodents, with a focus on the neuroretina and not the retinal arteries [1,4]. In these models, conflicting results have been reported, including upregulation, downregulation, and unaltered levels of PKC expression following ischemia [17–22]. We therefore believe that it is of major interest to map out these different intracellular signal transduction pathways in retinal ischemia, especially in the retinal arteries. For the present study, we chose to examine the PKCα, PKCβ1, and PKCβ2 isoforms in retinal ischemia. There are numerous isoforms of PKC, but PKCα, PKCβ1, and PKCβ2 are commonly studied isoforms in blood vessels when it comes to other ischemic conditions, such as stroke and ischemic heart disease [11,12]. These isoforms play a major role in regulating the development of these diseases. Furthermore, specific antagonists have been developed for these isoforms to hinder the injury associated with ischemia. PKCα, which is mainly expressed in the bipolar cells, seems to be the most abundant isoform in the retina, [23] while PKCβ has been proven to play a role in the development of diabetic retinopathy [15].

The aim of the present study was to perform a detailed delineation of the role of PKCα, PKCβ1, and PKCβ2 in retinal ischemia. We used a porcine eye model, which has a primate-like structure, as it is suitable for the separate analysis of the retinal arteries and the neuroretina. PKCα, PKCβ1, and PKCβ2 mRNA and protein expression were studied using real-time polymerase chain reaction (qRT–PCR), western blot analysis, and immunofluorescence staining.

## Methods

### Animals and anesthesia

A total of 28 domestic landrace pigs of both genders, with a mean bodyweight of 70 kg, were used for this study (Conventional pig breeder, Lund, Sweden). The night before the surgical procedure, food was withheld from the animals, but they were allowed free access to water. A 100 mg/ml intramuscular injection of ketamine (Ketaminol vet™; Farmaceutici Gellini S.p.A, Aprilia, Italy) per 15 mg/kg bodyweight, in combination with 20 mg/ml xylazine (Rompun vet™; Bayer AG, Leverkusen, Germany) per 2 mg/kg bodyweight, was used for premedication. Anesthesia was induced by continuous intravenous infusion of 20 mg/ml propofol (Diprivan™; Astra Zeneca, Södertälje, Sweden) at a dosage of 0.1–0.2 mg/kg/min in combination with intermittent fentanyl (Fentanyl B. Braun; B. Braun Melsungen AG, Melsungen, Germany) at approximately 3.5 μg/kg/h. The pigs had a mean arterial blood pressure of 92±7 mmHg. After completion of the experiments, animals were euthanized by a lethal intravenous injection of potassium 2 mmol/kg (ADDEX Potassium Chloride, Fresenius KABI SE, Uppsala, Sweden). All procedures and animal treatment took place in accordance with the guidelines of the Ethics Committee of Lund University, the Institute for Laboratory Animal Research (Guide for the Care and Use of Laboratory Animals), and the ARVO statement for the Use of Animals in Ophthalmic and Vision Research. The study was approved by the regional court under the hospice of the department of agriculture.

### Ischemia–reperfusion

During the surgery, ischemia was induced in one eye of each animal by raising the intraocular pressure (IOP), while the other eye was allowed to serve as a control. The posterior chamber of both eyes was cannulated with a 30 gauge needle. The IOP was raised to 80 mmHg in one eye by continuous infusion of balanced salt solution for ophthalmic irrigation (Amo^™^ Endosol^™^; AMO Groningen BV, Groningen, the Netherlands). The pressure was monitored using a Tono-Pen®XL tonometer (Medtronic, Jacksonville, FL). The control eye underwent the same surgical procedure but the pressure was not allowed to elevate. This eye will be referred to as the “sham-operated eye” in the text and figures. After 60 min, the cannulation needles were removed to allow reperfusion of the retinal vasculature. Ischemia was confirmed by indirect ophthalmoscopic examination by noting the blanching of retinal arteries. This was confirmed both directly after elevating the IOP, during ischemia (after 30 min) and at the end of the ischemic period (60 min).

### Tissue preparation

After 5 h, 12 h, or 20 h of reperfusion (7/14/22 h of anesthesia), both eyes of the pigs were enucleated, with the optic nerve included, during anesthesia. The eyes were dissected; the anterior segment and the vitreous humor were removed, and the eyecups were divided in half. One half was used for immunofluorescent staining, while the retina was dissected free from retinal pigment epithelium in the other half. Arteries were isolated from the neuroretina by careful dissection in a buffer solution at 4 °C (balanced salt solution for ophthalmic irrigation). The arteries were first to third-order branches. During the dissection of retinal arteries, blood was gently pushed out of the vessels. Central and peripheral pieces of each remaining neuroretina, devoid of major vessels, were collected and stored at −80 °C until used for qRT–PCR and western blot experiments. In the 12 h reperfusion group, some samples used in the qRT–PCR analysis included whole retinas, devoid of major vessels (n=8).

### RNA extraction and real-time polymerase chain reaction

RNA was extracted in two different ways. Samples from the sham-operated and the ischemia-reperfusion eyes of the same pig underwent the same RNA extraction procedure. Differences in mRNA were calculated in relative changes (the result from the ischemia-operated eye as a ratio of the sham-operated eye in the same pig). Taken this together we believe that the technique chosen for RNA extraction have not affected our results or conclusions. Using the first technique, we homogenized the tissue in 1 ml TRIzol (Invitrogen, Carlsbad, CA) using a metal ball and a TissueLyser (Retsch, Haan, Germany), according to the manufacturer’s instructions. Next, 200 µl chloroform was added to separate RNA from DNA, proteins, and cell debris. The homogenate was allowed to separate at room temperature before being centrifuged. The supernatant was transferred to new tubes and 500 μl isopropanol was added; the samples were then incubated at –20 °C overnight to allow precipitation of the RNA. Samples were centrifuged to further precipitate the RNA. The supernatant was removed and the RNA pellet washed once with 500 μl 75% ethanol. The supernatant was removed, the pellet dried, and then dissolved in Rnase-free water. Samples were incubated for 1 h on ice to allow the RNA pellet to dissolve completely. The light absorbance was measured at 260 nm and 280 nm using a spectrophotometer, and the RNA concentration and RNA/DNA ratio were recorded. The second technique was employed to extract RNA with an RNeasy Mini-kit (Qiagen, Valencia, CA), which allows simultaneous extraction of protein. Briefly, the tissue was homogenized in 600 μl RTL buffer using a metal ball and a TissueLyser, as described. The lysate was centrifuged to remove insoluble material, and the supernatant carefully transferred to a new tube. One volume of 70% ethanol was added, and the sample was then applied to an RNeasy mini-column and centrifuged. The flow-through was saved for protein extraction (see details as follows). The column was washed with RW1 buffer and RPE buffer, and the RNA eluted with 30 μl of Rnase-free water. The light absorbance was measured with a spectrophotometer, and the RNA concentration and RNA/DNA ratio recorded. From each eye, 4–8 μg total RNA was extracted from the retinal arteries, and 15–30 μg total RNA was extracted from the neuroretina.

Reverse transcription of total RNA to cDNA was performed using the GeneAmp RNA polymerase chain reaction kit in a Perkin-Elmer DNA Thermal Cycler (Perkin-Elmer Applied Biosystems, Foster City, CA). First-strand cDNA was synthesized from 1 μg total RNA in a 40 μl reaction using random hexamers as primers. The reaction was run at 42 °C for 90 min and thereafter at 72 °C for 10 min. qRT–PCR was performed in a GeneAmp 7300 Real Time PCR System using the GeneAmp SYBR® Green kit (Perkin-Elmer, Applied Biosystems) with the previously synthesized cDNA as template in a 25 μl reaction. A no-template control was included in all experiments. The GeneAmp 7300 system monitors the amplification of DNA in real-time using an optical imaging system, via the binding of a fluorescent dye to double-stranded DNA. Specific primers for porcine *PKCα*, *PKCβ1*, and *PKCβ2* are described in Table 1. The results were calculated relative to the amount of the housekeeping genes *β-actin* and elongation factor-1α (*EF-1α*), since these are continuously expressed at constant amounts in cells [24]. The primer sequences for these housekeeping genes are given in Table 1.

**Table 1:** Real-time PCR primers.

**Gene name**	**GenBank number**	**Sequence (5′-3′)**
*PKCα*	AY093442	F: AACAAGGCTTCCAGTGCCAA
		R: GAACTCATGGCACCTCTTGTGA
*PKCβ1*	AY093443	F: ACGAATTTGCTGGCTTCTCC
		R: TGGCCTGAAGTCTTACACTCCA-3′
*PKCβ2*	AY093444	F: GCTGTGTAGATCTCCGTCCTTCAT
		R: AGGTCACCACAATAGCTGTCGA
*β-actin*	U07786	F: CCTTCAACTCGATCATGAAGTGC
		R: CGTAGAGGTCCTTCCTGATGTCC
*EF-1α*	AM040195	F: GCTGACTGTGCTGTCCTGATTG
		R: TGTAGGCCAGAAGAGCATGCT

Gene name, GenBank number and primer sequence for primers used in real-time PCR experiments.

The primers were dissolved in water according to the manufacturer’s instructions, and a mixture of reverse and forward primers was made. qRT–PCR was performed with the following profile: 1 cycle of 50 °C for 2 min, and 95 °C for 10 min followed by 40 cycles of 95 °C for 15 s, and 60 °C for 1 min. This was followed by dissociation, 1 cycle of 95 °C for 15 s, 60 °C for 30 s and 95 °C for 15 s. To check that the cDNA levels of *β-actin*, *EF-1α*, and *PKC*s were amplified at the same efficiency during qRT–PCR, we constructed a standard curve in which the values of C_T_ were plotted against the cDNA concentration on the basis of the following equation:

$$C_{T}={\left[\text{log}\left(\text{1}+E\right)\right]}^{-\text{1}}\text{ log }\left(\text{concentration}\right),$$

where *E* is the amplification efficiency, with the optimal value of 1. The amount of *PKC* mRNA in the specimens was calculated relative to the amount of *β-actin* and *EF-1α* mRNA in the same sample using the relation,

$$X_{0}/R_{0}={\text{2}}^{CTR\mathrm{-C}TX}$$

where *X*_0_ equals the original amount of *PKC* mRNA, *R*_0_ equals the original amount of *β-actin* mRNA, *C_TR_* is the *C_T_* value for *β-actin*, and *C_TX_* is the *C_T_* value for *PKC*.

### Protein extraction and protein content determination

The flow-through was collected from RNA extraction and incubated with 4 volumes of ice-cold acetone at −20 °C for 30 min. The samples were then centrifuged for 10 min at 16,000 xg, at 4 °C and the supernatant discarded. The protein pellet was air-dried and resuspended in 8 M urea. The total protein concentration was determined using a BioRad DC kit (BioRad, Hercules, CA) and measurement of the absorbance at 750 nm on a microplate photometer (Thermo, Waltham, MA). Protein samples were used immediately for western blot analysis or stored at −80 °C until use. From each eye, 100–200 μg protein was extracted from the retinal arteries, and 1–2 mg protein was extracted from the neuroretina.

### Western blot

Proteins of interest were evaluated in the neuroretina and retinal arteries separately. Protein samples were mixed with NuPAGE LDS sample buffer (Invitrogen) and boiled for 5 min. Equal amounts of protein (30 μg/lane for neuroretina and 20 μg/lane for retinal arteries) were loaded onto a NuPAGE 4%–12% Bis-Tris Gel (Invitrogen) and separated by SDS–PAGE. A molecular weight marker (SeeBlue® Plus2; Invitrogen) was loaded onto each gel for protein band identification. After separation, the proteins were transferred to a nitrocellulose membrane (GE Osmonics, Minnetonka, MN). The membrane was subsequently blocked with 6.5% nonfat milk in PBS (0.14 M NaCI, 0.01 M PO_4_ Buffer, 0.003 M KCI, pH 7.45) overnight at 4 °C and washed for three times with 0.1% Tween-PBS (T-PBS) for 15 min each time. The membranes were then incubated overnight at 4 °C with the primary antibodies of interest: 1:1,000 mouse anti-PKCα (Nordic BioSite, Täby, Sweden), 1:500 rabbit polyclonal anti-PKCβ1 (Nordic BioSite), 1:500 rabbit monoclonal anti-PKCβ2 (Nordic BioSite), 1:1,000 rabbit polyclonal phosphospecific anti-PKCα (Biosource, Camarillo, CA), 1:1,000 rabbit polyclonal phosphospecific anti-PKCβ2 (Biosource), or 1:5,000 mouse monoclonal β-actin (Santa Cruz Biotechnology, Santa Cruz, CA). Incubation was followed by washing three times with T-PBS for 15 min each time. The membranes were then incubated for 4 h at room temperature with the appropriate secondary antibody: 1:500 swine polyclonal anti-rabbit IgG-horseradish peroxidase or 1:500 rabbit polyclonal anti-mouse IgG-horseradish peroxidase (Dako, Glostrup, Denmark). Membranes were then washed three times with T-PBS for 15 min each time. Levels of β-actin were used to confirm equal loading of the lanes. The membranes were developed using Amersham ECL Plus Western Blotting Detection Reagents (GE Healthcare, Buckinghamshire, UK) and visualized using a Fujifilm LAS-1000 Luminescent Image Analyzer (Fujifilm, Stamford, CT).

### Immunofluorescence staining

Each half of both eyes were fixed in 4% paraformaldehyde for 5 h. After fixation, the tissue was rinsed in 0.1 M Sørensen’s phosphate buffer (28 mM NaH_2_PO_4_ and 72 mM Na_2_HPO_4_; pH 7.2), and thereafter washed in the same solution with increasing concentrations of sucrose (5% to 25%). The specimens were embedded in 30% egg albumin and 3% gelatin and were stored at –80 °C until sectioning. They were serially sectioned at 12 µm in a cryostat (Microm HM500M; Thermo Scientific, Walldorf, Germany) and placed on microscope slides (Menzel, Braunschweig, Germany), three sections on each slide. The slides were allowed to dry at room temperature for 30 to 60 min, and were then stored at –20 °C until further use.

Anti-PKC sections were permeabilized in a mixture of PBS and 0.25% Triton X-100 for 15 min then blocked in PBS,1% BSA, and 5% normal serum for 1 h at room temperature. Specimens were incubated overnight at 4 °C with 1% BSA and 2% normal serum and the primary antibody of interest: 1:200 rabbit polyclonal phosphospecific anti-PKCα (Biosource), 1:100 rabbit polyclonal phosphospecific anti-PKCβ1 (Biosource), 1:200 rabbit polyclonal phosphospecific anti-PKCβ2 (Biosource), 1:10 mouse monoclonal anti-CD31 (AbD Serotec, Oxford, UK), and 1:200 mouse monoclonal antismooth muscle actin (Santa Cruz Biotechnology). CD31, also known as PECAM-1, is expressed by various cell types, but particularly by endothelial cells [25]. Smooth muscle actin is commonly used for detection of smooth muscle tissue. Sections were washed with PBS buffer and incubated with the appropriate secondary antibody, 1:200 fluorescein isothiocyanate goat anti-rabbit (Cayman Chemicals, Ann Arbor, MI) for localization of phosphospecific anti-PKCβ1 and PKCβ2 in the neuroretina and 1:50 fluorescein isothiocyanate swine anti-rabbit (Dako) as well as 1:200 Texas Red donkey anti-mouse (Jackson ImmunoResearch, West Grove, PA) on all other samples for 1 h at room temperature. After an additional wash with PBS buffer, the slides were mounted in anti-fading mounting medium (Vectashield; Vector Laboratories Inc., Burlingame, CA). In the present study, the immunofluorescence technique for localization of protein expression was only used for examining the phosphorylated forms of PKC. The non-phosphorylated forms of specific PKC isoenzymes were expected to be expressed in the same cells as the phosphorylated forms of the same PKC isoenzyme. Quantification of both phosphorylated and total PKC was performed using western blot.

Vertical sections including the optic nerve head were examined at the central part of the retina from four pigs in the group subjected to ischemia and 20 h of reperfusion. Costaining with PKC and CD31 or smooth muscle actin was done in sections from one to two pigs. The staining intensity was viewed with a light microscope equipped for fluorescence microscopy (Zeiss Axiophoto; Carl Zeiss, Oberkochen, Germany), and photographs were taken with an attached digital camera (Zeiss AxioCam). For the purpose of staining intensity comparisons, sections from ischemia-reperfusion eyes and corresponding controls were processed at the same time to minimize variability.

### Statistical analysis

Statistical analysis was performed using paired Student’s ratio *t*-test and GraphPad 5.0 software. Correction for multiple comparisons was performed manually using Bonferroni correction, which is calculated by multiplying the p-values with the number of performed analysis. Different primers and antibodies were considered separate analysis, and no correction was made between them. Exact p-values (after Bonferroni correction) are given in the text and figures. Values are presented as means±the standard error of the mean (SEM).

### Limitations

The amount of protein and mRNA that can be extracted from the retinal arteries is less than what can be extracted from the neuroretina. Since the amount of material was limited, we focused on analyzing the phosphorylated forms of PKC in both the retinal arteries and the neuroretina, while the total PKC were only analyzed in the neuroretinal samples. For technical reasons, we were unable to obtain any results from the protein analysis of samples from eyes exposed to ischemia and 5 h of reperfusion. For ethical reasons, we chose not to sacrifice another six pigs for these experiments only.

## Results

### Real-time PCR

**Figure 1:**
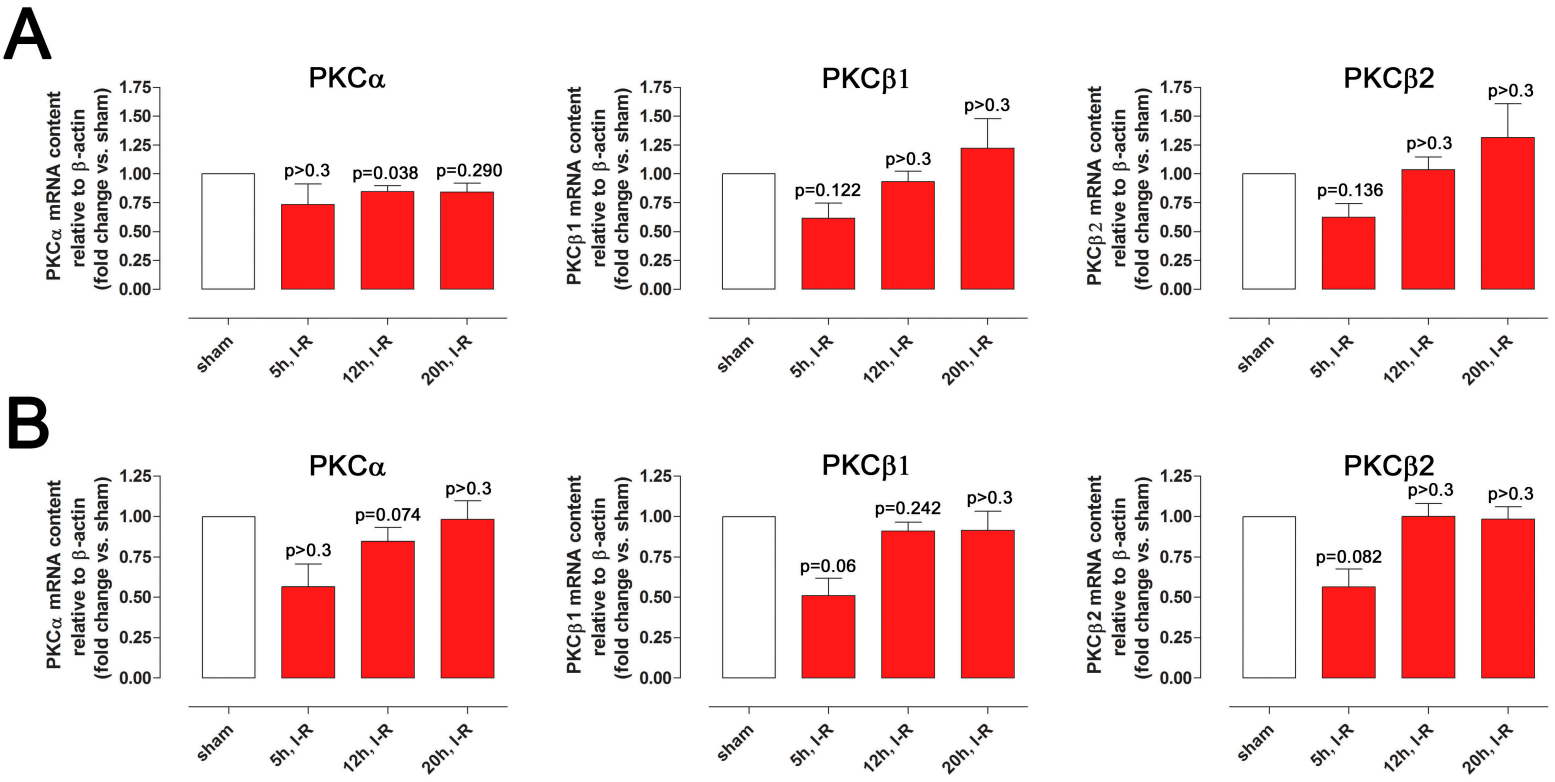
PKC mRNA levels in the retinal arteries and neuroretina. *PKCα*, *PKCβ1*, and *PKCβ2* mRNA expression levels assessed by real-time PCR in (**A**) the retinal arteries and (**B**) in the neuroretina in eyes subjected to ischemia and 5 (n=6), 12 (n=14), or 20 (n=6) hours of reperfusion versus sham-operated eyes. Values are presented as mean±SEM. Statistical comparison was performed using Student’s paired ratio *t*-test (ischemia–reperfusion versus sham-operated) with Bonferroni correction. Exact p-values are given in the figure. Note that the levels for *PKCα*, *PKCβ1*, and *PKCβ2* show similar patterns of change in the retinal arteries and the neuroretina.

*PKCα*, *PKCβ1*, and *PKCβ2* mRNA levels tended to be lower in the retinal arteries and in the neuroretinas from the eyes exposed to ischemia followed by 5 h, 12 h, and 20 h of reperfusion compared to sham-operated eyes (Figures 1A,B); however these differences did not reach statistical significance (for exact p-values, see Figures 1A,B).

Similar patterns of *PKCα*, *PKCβ1*, and *PKCβ2* mRNA expression were seen when using *β-actin* as the reference gene or when compared to *EF-1α* (data not shown), indicating that these genes were reliable references. The standard curves for each primer pair had similar slopes (3.4 for *PKCα*, 3.3 for *PKCβ1*, 3.3 for *PKCβ2*, and 3.3 for *β-actin*), suggesting that the *PKCα*, *PKCβ1*, *PKCβ2*, and *β-actin* cDNA were amplified with similar efficiency. The value of each slope was close to 3.3, and the amplification efficiencies were close to 1.0, which is optimal.

### Western blot

**Figure 2:**
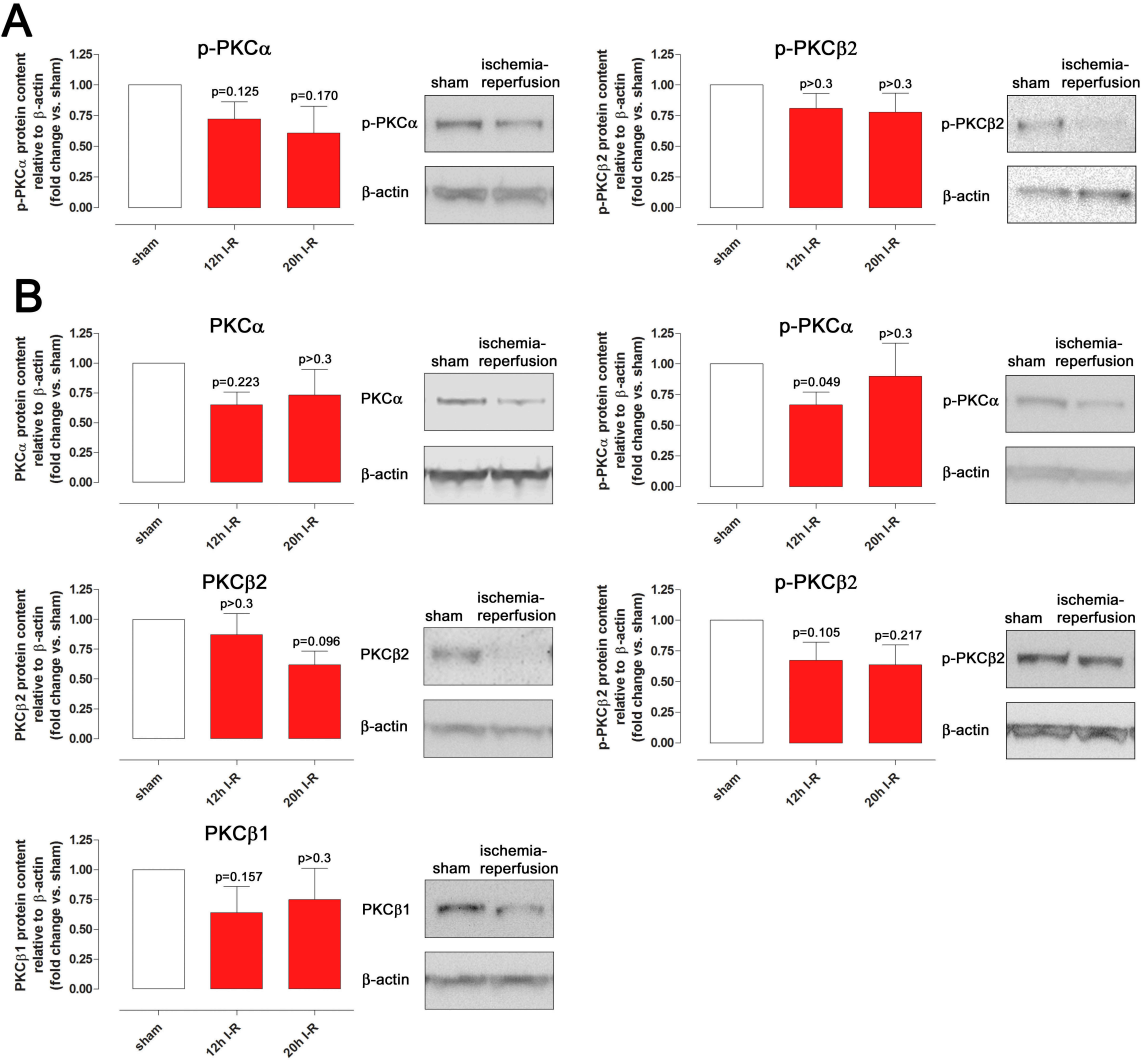
PKC protein levels in the retinal arteries and neuroretina. Phosphorylated and total PKCα, PKCβ1, and PKCβ2 protein expression levels, assessed by western blot, in (**A**) retinal arteries and (**B**) neuroretina, in eyes subjected to ischemia and 12 (n=7) or 20 (n=5) hours of reperfusion versus sham-operated eyes. The right panels are representative examples of western blots of neuroretina and retinal arteries from animals in the 20 h of reperfusion group. Values are presented as mean values±SEM. Statistical comparison was performed using Student’s paired ratio *t*-test (ischemia–reperfusion versus sham) with Bonferroni correction. Exact p-values are given in the figure.

The protein levels of phosphorylated PKCα and PKCβ2 tended to be lower in the retinal arteries from the eyes subjected to ischemia followed by 12 h and 20 h reperfusion, compared to the sham-operated eyes (Figure 2A). These differences did not reach statistical significance. Similar patterns of change were also seen for the neuroretina (Figure 2B). The total PKCα, PKCβ1, and PKCβ2 in the neuroretina were slightly lower following ischemia-reperfusion (Figure 2B). Phosphorylated PKCβ1 only gave weak bands on western blot and were not sufficient for reliable quantitative analysis.

### Immunofluorescence

**Figure 3:**
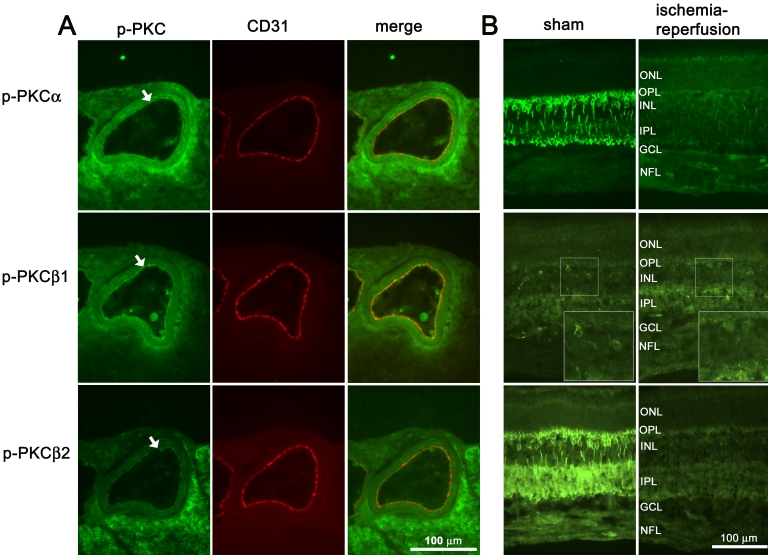
PKC immunoreactivity in the retinal arteries and neuroretina. Representative examples showing phosphorylated PKCα, PKCβ1, and PKCβ2 immunoreactivity in the retinal arteries and retina following ischemia and 20 h of reperfusion. **A:** Double staining with CD31 (also called PECAM-1), an endothelial cell marker, showed co-localization of phosphorylated PKC in the endothelium (arrows). Weak phosphorylated PKC staining could also be seen in the smooth muscle layer. **B:** The lower levels of PKCα, PKCβ1, and PKCβ2 observed in the neuroretina after ischemia–reperfusion, according to western blot, were reflected in the immunofluorescence staining results, showing less staining for phosphorylated PKCα and PKCβ2 in the ischemia–reperfusion eyes compared to sham-operated eyes. Furthermore, the phosphorylated PKCβ1 staining showed fewer labeled bipolar cells bodies in the eyes subject to ischemia-reperfusion compared to sham-operated eyes (see insert in the p-PKCβ1 picture). Similar results were seen in all pigs studied. Abbreviations: outer nuclear layer (ONL), outer plexiform layer (OPL), inner nuclear layer (INL), inner plexiform layer (IPL), ganglion cell layer (GCL), and nerve fiber layer (NFL).

**Figure 4:**
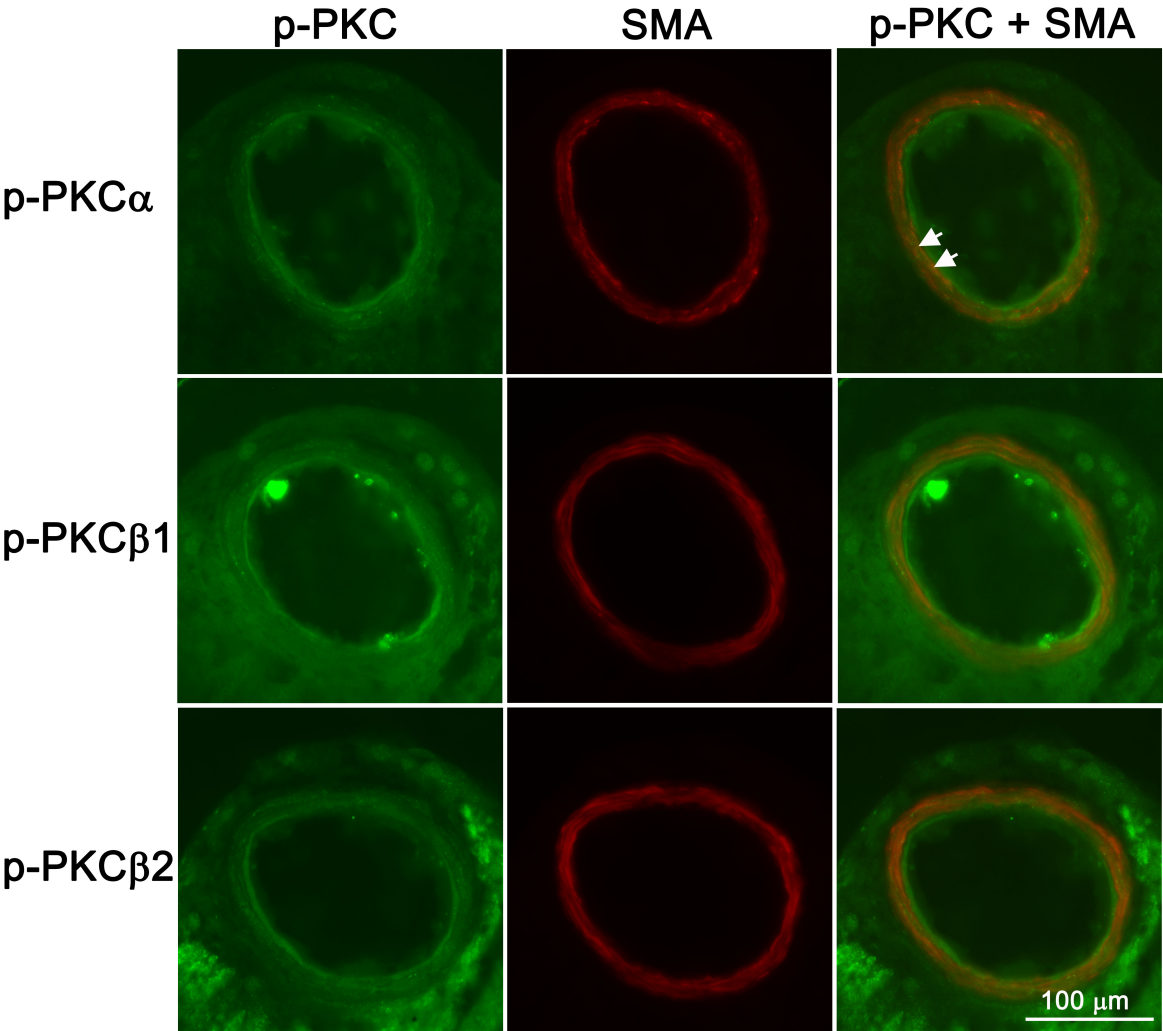
PKC immunoreactivity in the smooth muscle layer of the retinal arteries. Representative examples showing phosphorylated PKCα, PKCβ1, and PKCβ2 immunoreactivity in the retinal arteries following ischemia and 20 h of reperfusion. Double staining with smooth muscle actin, a smooth muscle marker, showed colocalization with phosphorylated PKC in the smooth muscle layer (arrows). Note that the colocalization was most apparent for phosphorylated PKCα.

In the retinal arteries, immunofluorescence staining for phosphorylated PKCα, PKCβ1, or PKCβ2 was primarily localized to the endothelial cells (Figure 3A). The endothelium was visualized by staining with the endothelial cell marker, CD31. Weak staining was also seen at a lower degree in the smooth muscle layer of the blood vessels, this was most apparent for phosphorylated PKCα (Figure 4).

In the neuroretina the staining was localized to the bipolar cells (Figure 3B). Lower phosphorylated PKCα and PKCβ2 staining intensity was observed in the neuroretina in the ischemia-reperfusion eyes compared to the control eyes. Furthermore, the phosphorylated PKCβ1 staining showed fewer labeled bipolar cells bodies in the eyes subject to ischemia-reperfusion compared to sham-operated eyes (see insert in the p-PKCβ1 picture).

## Discussion

Retinal ischemia is due to circulatory failure, which has its origin in the vasculature. Most studies performed so far have focused on identifying neuroprotective agents for the treatment of retinal ischemia-reperfusion injury. The retinal blood vessels are key organs in circulatory failure. Therefore, our aim was to study the retinal vasculature separately from the neuroretina, to identify the intracellular signal-transduction pathways, specifically PKC, involved in the development of injury following retinal circulatory failure.

Immunofluorescence staining verified the presence of phosphorylated PKCα, PKCβ1, and PKCβ2 in retinal arteries. The staining was primarily localized to the endothelial cells. This is, to the best of our knowledge, the first analysis of PKC localization in the retinal vasculature following ischemia-reperfusion injury. The mRNA levels for *PKCα*, *PKCβ1*, and *PKCβ2* were clearly lower in the retinal arteries from the ischemia-reperfusion eyes than from the sham-operated fellow eyes. Also, the protein levels for phosphorylated PKCα and PKCβ2 tended to be lower following ischemia-reperfusion, although these results did not reach statistical significance. Unlike the retinal blood vessels, the vasculature has been thoroughly analyzed with regard to ischemic conditions in other organs. The level of PKC is changed in the vasculature of the brain during stroke, in coronary arteries during ischemic heart disease, and in several tissues due to diabetes [12,26,27]. Indeed, PKC inhibitors have been shown to prevent the development of pathological receptor expression in the vascular wall and decrease the extent of stroke injury following middle cerebral artery occlusion and subarachnoidal hemorrhage in the rat [12,13]. The development of pathological receptor expressions in coronary arteries has also been found to be inhibited by PKC antagonists [14].

The staining intensity for phosphorylated PKCα, PKCβ1, and PKCβ2 was especially prominent in bipolar cells in the neuroretina. The occurrence of PKC isoforms has been thoroughly investigated in a variety of animal species, and studies in mammals verify the presence of immunofluorescence staining for PKCα and PKCβ in bipolar rod cells of the neuroretina [28]. In the present study, the levels of PKCα, PKCβ1, and PKCβ2 mRNA, and protein expression in the neuroretina were lower in eyes subjected to ischemia-reperfusion than in sham-operated eyes. Previous studies, using a variety of ischemic models, have reported conflicting results concerning the effects of retinal ischemia on PKC expression in the neuroretina [20–22]. The reason for this discrepancy may be due to the type and severity of ischemic insult, as well as the animal model studied. Similar downregulation of the immunoreactivity of PKCα after ischemia, as observed in the present study, has been reported in the rabbit retina [21].

It cannot be deduced from the present study whether it is the ischemia alone or the reperfusion that triggers the PKC alterations. However, it is generally believed that it is both the ischemia and the following reperfusion that trigger changes seen after ischemia-reperfusion injury [1]. The mechanism underlying the lower PKC mRNA and protein levels is not known, but it may be related to decreased transcription and translation of PKC, triggered by humoral factors that are changed during ischemia. It is believed that calpain, a Ca^2+^-dependent protease, may be responsible for the proteolysis of certain PKC isoforms including PKCβ following ischemia-reperfusion in the brain [29]. Also, dephosphorylation of the PKC protein may render PKC more sensitive to proteolysis. PKC inhibitors have been shown to prevent the development of injury in the heart and brain in animal models following an ischemic event [10,12]. However, the effect of the PKCβ-specific inhibitor, LY333531, has been investigated in experimental research and clinical trials for the treatment of retinal vascular diseases, including retinal vein occlusion and diabetic retinopathy, but the results have not been as promising as hoped [16,30,31]. Knowledge of the role of PKC in retinal ischemia is still fairly limited, and whether the function of the PKC signaling pathway is impaired or amplified during ischemic injury is remains unknown due to conflicting reports.

We wanted to monitor the changes in intracellular signal-transduction pathways, in this case PKC, during the development of tissue injury following retinal ischemia. Therefore, the retina was examined following different durations of reperfusion after the ischemic event. Studying different durations of reperfusion may provide insight into both the initial molecular intracellular events and the ensuing tissue injury. The present study focused on the initial events. At 5 h of reperfusion, PKCα, PKCβ1, and PKCβ2 expression levels were lower in the ischemia-reperfusion eyes than in the sham-operated eyes. After longer duration of reperfusion (12 and 20 h) there was no apparent difference in the expression of PKC levels between the ischemia-reperfusion and sham-operated eyes. This pattern of change may reflect initial injury to the tissue and recovery, with PKC levels returning to baseline. Nevertheless, it cannot be deduced from the present study whether a downregulation of PKC is protective, or is part of the detrimental process of tissue injury.

The porcine eye has a typical primate-like architecture, including retinal blood vessels, which are useful for experimental analysis [5–7]. Retinal ischemia was induced by raising the IOP for 60 min. This time period is commonly used for inducing high IOP ischemia-reperfusion. High IOP ischemia-reperfusion is a frequent model for experimental retinal ischemia research [1] and has been described in several species including rats and rabbits. High IOP produces global ischemia, with obstruction of both the retinal and uveal circulation, whitening of the fundus, and iris pallor. The method is known to produce pathological features similar to that seen after central retinal artery occlusion [1]. Siliprandi et al. [32,33] showed in cats that the retinal injury after elevating the IOP is caused by ischemic insult and is not the result of increasing the pressure per se. In the present study, blanching of the arteries and a pale retina were noted by indirect ophthalmoscopy using an IOP of 80 mmHg, which would suggest that this level of IOP was sufficient to cease blood flow. One limitation of the present study was that the oxygen tension was not measured in the retina. It has been shown that retinal oxygen tension may be retained also at high IOP in the pig retina [34] as a consequence of autoregulation. In the present study there were some variations in the results. It cannot be ruled out that this autoregulation accounts for at least some of the variability seen in our results. Also, interindividual variations in the resistance to an ischemic insult have been reported before and may account for some of the variance of the results [1].

In the present study, the retinal arteries were dissected free from the neuroretina and analyzed separately. It is possible there may have been some slight contamination of the retinal arteries with neuroretina and vice versa. Unfortunately, this cannot be prevented. Contaminants from the neuroretina may be removed from the retinal arteries by using digestive enzymes (e.g., trypsin) or osmotic shock (e.g., distilled water) [35]. However, osmotic shock is primarily a method in which arteries and veins are extracted together. Furthermore, trypsin-treated samples cannot be used for real-time PCR and western blot experiments.

In conclusion, the blood vessels of the retina are key in circulatory failure, and we therefore analyzed the retinal arteries separately from the neuroretina. The levels of PKC mRNA and protein were lower in both the retinal arteries and the neuroretina from eyes subjected to ischemia-reperfusion than in sham-operated eyes. It remains unclear whether PKC is involved in cell-survival signaling or mediates detrimental processes. The present study adds to the knowledge about the signal-transduction pathways involved in the development of retinal injury following ischemia. This information may aid in the identification of new targets for pharmacological treatment.
